# Examining the associations between manic symptoms and cognitive performance in bipolar disorders: evidence from a cross-sectional replication study in the FACE-BD cohort

**DOI:** 10.1186/s40345-026-00420-2

**Published:** 2026-04-15

**Authors:** Nathan Vidal, Eric Brunet-Gouet, Solène Frileux, Raoul Belzeaux, Philippe Courtet, Thierry D’Amato, Caroline Dubertret, Bruno Etain, Emmanuel Haffen, Dominique Januel, Marion Leboyer, Antoine Lefrere, Pierre-Michel Llorca, Emeline Marlinge, Paula Martinez, Katia M’Bailara, Emilie Olié, Mircea Polosan, Raymund Schwan, Michel Walter, the FACE-BD (FondaMental Academic Centers of Expertise for Bipolar Disorders) group*, E Olié, M Leboyer, E Haffen, P.M Llorca, V Barteau, S Bensalem, O Godin, H Laouamri, K Souryis, S Hotier, A Pelletier, N Drancourt, J.P Sanchez, E Saliou, C Hebbache, J Petrucci, L Willaume, E Bourdin, F Bellivier, M Carminati, E Marlinge, J Meheust, V Hennion, A Richard, B Aouizerate, N Da Ros, A Desage, C Elkael, S Gard, F Hoorelbeke, K M’bailara, I Minois, J Sportich, L Boukhobza, M Benramdane, P Courtet, B Deffinis, S Denat, D Ducasse, M Gachet, F Molière, L Nass, G Tarquini, A Lefrere, M Cermolacce, E Moreau, F Groppi, L Lescalier, J Pastol, H Polomeni, J Baurberg, R Cohen, G Gross, R Schwan, T Schwitzer, O Wajsbrot-Elgrabli, T Bougerol, B Fredembach, Q Denoual, A Bertrand, A Pouchon, M Polosan, L Brehon, L Durand, V Feuga, S Frileux, P Roux, E Vaux, V Aubin, I Cussac, M.A Dupont, J Loftus, I Medecin, C Dubertret, N Mazer, C Portalier, C Scognamiglio, A Bing, P Laurent, L Samalin, L Foures, D Lacelle, S Pires, C Doriat, O Blanc, D Bennabi, M Nicolier, Christine Passerieux, Paul Roux

**Affiliations:** 1 Fondation FondaMental, Créteil, France; 2 DisAP, MOODS Team, INSERM UMR1018, CESP, Université de Versailles Saint-Quentin-En-Yvelines - Université Paris-Saclay, Le Chesnay, France; 3 Centre Hospitalier de Versailles, Service Universitaire de Psychiatrie d’Adultes et d’Addictologie, Le Chesnay, France; 4 CHU Montpellier, Montpellier, France; 5 IGF, University of Montpellier, CNRS, INSERM, Montpellier, France; 6 Department of Emergency Psychiatry and Acute Care, Lapeyronie Hospital, CHU Montpellier, Montpellier, France; 7 INSERM U1028, CNRS UMR5292, University Lyon 1, Villeurbanne, France; 8 Lyon Neuroscience Research Center, Psychiatric Disorders: From Resistance to Response Team, Lyon, France; 9 Faculté de Médecine, Université de Paris Inserm UMR1266, Sorbonne Paris Cité, Paris, France; 10 AP-HP, Groupe Hospitalo-Universitaire AP-HP Nord, DMU ESPRIT, Service de Psychiatrie et Addictologie, Hôpital Louis Mourier, Colombes, France; 11 Université Paris Cité, INSERM, Optimisation Thérapeutique en Neuropharmacologie, OPTEN U1144, 75006 Paris, France; 12 Département de Psychiatrie et de Médecine Addictologique, Hôpitaux Lariboisière-Fernand Widal, GHU APHP.Nord - Université Paris Cité, F-75010 Paris, France; 13 Université Marie et Louis Pasteur, UMR INSERM 1322 LINC, Besançon, France; 14 Service de Psychiatrie de l’adulte, CIC-1431 INSERM, CHU de Besançon, F-25000 Besançon, France; 15 Université Sorbonne Paris Nord, 93017 Bobigny Cedex, France; 16 Département de Recherche Clinique, Pôle Universitaire 03 G03, EPS Ville Evrard, Neuilly sur Marne, France; 17 Université Paris Est Créteil (UPEC), Inserm U 955, Neuropsychiatrie Translationnelle, DMU IMPACT (AP-HP), Département de Psychiatrie, Fondation FondaMental, Créteil, France; 18 Pôle de Psychiatrie, Assistance Publique Hôpitaux de Marseille, Marseille, France; 19 NT-UMR7289, CNRS Aix-Marseille Université, Marseille, France; 20 Centre Hospitalier et Universitaire, Département de Psychiatrie, Université d’Auvergne, EA 7280, Clermont-Ferrand, France; 21 Pôle de Psychiatrie, Centre Hospitalier Princesse Grace, Monaco, Monaco; 22 Centre Hospitalier Charles Perrens, Pôle de Psychiatrie Générale et Universitaire, Bordeaux, France; 23 Univ. Bordeaux, LabPsy, UR 4139, F-33000 Bordeaux, France; 24 Univ. Grenoble Alpes, Inserm, U1216, CHU Grenoble Alpes, Grenoble Institut Neurosciences, Grenoble, France; 25 Université de Lorraine, Centre Psychothérapique de Nancy, Inserm U1254, Nancy, France; 26 Service Hospitalo-Universitaire de Psychiatrie Générale et de Réhabilitation Psycho Sociale 29G01 et 29G02, CHRU de Brest, Hôpital de Bohars, Brest, France; 27 Service de Psychiatrie du Secteur 78G18, Centre Hospitalier de Plaisir, Plaisir, France

## Abstract

Studies examining the effect of mania on cognition in Bipolar Disorders (BD) have yielded contradictory results. Koenders and collaborators showed in 2014 that, among 189 adults with BD, adults with subclinical manic symptoms perform a task measuring divided attention significantly better than adults with less or more severe manic symptoms. We aimed to replicate this finding in a larger sample. We included adults from the FACE-BD cohort with a BD diagnosis based on the DSM-IV-R criteria. We excluded adults with a current characterized depressive or manic episode or with other possible sources of cognitive impairment. We assessed the associations between the YMRS score and cognitive functions involved in divided attention using linear regression models, adjusting for the same covariates as the original study. We found no significant linear or quadratic associations between the YMRS total score and attention, working memory, and executive performance in the bivariable models nor after adjusting for covariates in 2,739 adults with BD. We were unable to replicate the findings reported by Koenders and collaborators. The low variance and mean severity of manic symptoms in our sample may partly explain the absence of significant associations. Our results suggest that subclinical hypomanic symptoms neither enhance nor impair cognitive performance in adults with BD, calling into question the benefit of residual hypomanic symptoms on patients’ cognitive functioning.

**Supplementary Information:**

The online version contains supplementary material available at 10.1186/s40345-026-00420-2.

## Introduction

In bipolar disorders (BD), studies examining the effect of mania on cognition have yielded contradictory results. One cross-sectional study did not detect any significant differences in cognitive performance between 34 manic/hypomanic and 44 euthymic adults, though manic/hypomanic adults performed worse on certain tests of executive function and attention (Martínez-Arán et al. 2004). However, a meta-analysis based on studies including 91 to 140 adults reported that adults experiencing a manic or mixed episode had significant impairments in verbal learning, attention, memory and executive function compared to euthymic patients, even in the absence of psychotic symptoms (Kurtz and Gerraty 2009). Koenders and collaborators (2014) hypothesized that such inconsistencies may be explained by a nonlinear relationship between manic symptoms and cognitive performance. They found a significant quadratic relationship between Young Mania Rating Scale (YMRS) scores and divided attention, measured by the Test for Attentional Performance (TAP) in adults with BD: adults with YMRS scores lower than 3 or higher than 13 had significantly poorer cognitive performance than adults with YMRS scores between 4 and 12 (Koenders et al. 2014). A YMRS score higher than 14 conventionally indicates a characterized manic episode. Their results suggest that residual manic symptoms may improve divided attention. However, the *p*-value of the association was close to the 5% level, indicating high risk of false positives. Besides, one study reported a significant linear association between the severity of residual manic symptoms measured by the Bech-Rafaelsen Mania Scale (BRMS), and poorer attention (unstandardized *β* = −0.246, *p* = 0.023) in 57 euthymic patients with a total BRMS score < 6 (Zhang et al. 2019). To better understand the association between manic symptoms and divided attention, we aimed to replicate the design used by Koenders and collaborators, testing the quadratic associations between YMRS scores and performance across attention, working memory, and executive function, which relate to divided attention, in a larger sample of patients.

## Methods

We included patients from the FACE-BD (FondaMental Advanced Centers of Expertise for Bipolar Disorders) cohort, recruited by a French national network established by the *FondaMental* Foundation (https://www.fondation-fondamental.org). The diagnosis of BD was determined using the Structured Clinical Interview for DSM-IV-R criteria. We included outpatients aged 18 and older with type I, type II, or not otherwise specified bipolar disorder. We excluded people with neurological comorbidities as in the original study. Additionally, we excluded patients with a MADRS score > 10 and patients with a current characterized depressive or manic episode according to the DSM.

We measured the severity of hypomanic symptoms with the YMRS (Young et al. 1978), and performance in attention, working memory, and executive function. Attention, working memory, and executive function performance were assessed with various neuropsychological tests, administered by experienced neuropsychologists:

- Attention, evaluated through Conners’ Continuous Performance Test II V.5, analyzing omission, commission, variability, and detectability scores.
- Working memory, assessed with WAIS Digit Span (total score) and Spatial Span (forward and backward scores) from the Wechsler Memory Scale, version III.
- Executive function, measured using the Stroop test (color/word condition), semantic and phonemic verbal fluency, and Trail Making Test, Part B.

We also assessed other cognitive domains:

- Verbal and perceptual reasoning, assessed with WAIS Vocabulary and Matrices.
- Processing speed, assessed using Digit Symbol Coding (WAIS-III) or Coding (WAIS-IV), WAIS Symbol Search, and the Trail Making Test, Part A.
- Verbal memory, measured by the California Verbal Learning Test, including short and long delay-free recall and total recognition.

Raw scores for each test were converted to standardized scores using age and sex-matched norms. Scores from WAIS-III and WAIS-IV tests were standardized separately before being pooled together. We computed a global cognitive score by averaging the standardized scores across all cognitive domains.

We recorded patients’ sex at birth (self-reported), and education level, the use of benzodiazepines, antidepressants, lithium, other mood stabilizers, antipsychotics, and age during the visit. We also collected the BD subtype, and any symptom of substance use disorders within the month prior the visit. Unlike the original study, we additionally collected covariates that are expected to be associated with cognition and mood: history of psychosis, anticholinergic burden measured using a suited anticholinergic burden scale (Vidal et al. 2023), the duration of illness, the type of WAIS used for working memory assessment (1 indicating the WAIS-IV was used, 0 if the WAIS-III was used), the previous number of manic episodes, and a binary variable indicating whether the last manic episode occurred within three months before the visit.

Statistical analyses were performed using *R* version 4.3.1. We hypothesized that data was Missing At Random (MAR) and estimated it using multivariate imputation by chained equations, except for attention performance, which had more than 30% missing data. We assessed the associations between the YMRS score and attention, executive functions, and working memory scores using bivariable linear regressions with simple terms. We then included a quadratic term (i.e., squared YMRS total score) in the models, and adjusted for the same covariates included in the original study by Koenders and collaborators, except for age and sex, which were already accounted for by cognitive standardization.

## Results

**Fig. 1:**
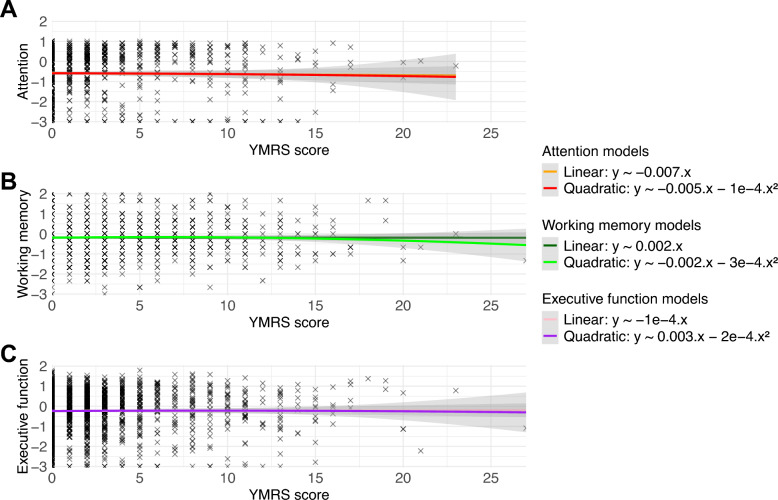
Distribution of YMRS scores across attention **(A)**, working memory **(B)** and executive function **(C)**. We represented the curves of estimated bivariable models with and without quadratic terms

We included 2,087 adults with BD (61.1% females, aged 40.4 ± 13.5), with a mean YMRS total score of 2.2 ± 3.6, ranging between 0 and 27 (Table 1). Our sample included 52.1% of adults with type I BD and 38.4% with type II BD. We found no significant linear or quadratic associations between the YMRS total score and attention, executive function, or working memory performance at the 5% level nor in the bivariable models nor after adjusting for the same set of covariates as Koenders and collaborators (Tables 2, 3, and 4). Graphical representations of the associations between the YMRS total score and each cognitive domain are displayed in Fig. 1. The associations between YMRS and attention, executive function, and working memory, remained non-significant after adjusting for additional covariates such as history of psychosis, anticholinergic burden, the duration of illness, the type of WAIS used for working memory assessment, the previous number of manic episodes, and a binary variable indicating whether the last manic episode occurred within three months before the visit (supplementary material SM1). The associations between YMRS and attention, executive function, and working memory, remained non-significant after the exclusion of individuals aged > 65 years (supplementary material SM2) or with extreme global cognitive performance (e.g., higher or lower than two standard deviations of the sample) (supplementary material SM3). We also investigated the effects of each individual YMRS item. The item of the YMRS assessing sexual interest showed a significant negative linear association and a positive quadratic associations (“U-shaped”) with attention performance in multiple linear regression models but the significance did not persist after correcting for multiple comparisons using the false-discovery rate by Benjamini-Hochberg (Benjamini and Hochberg 1995) (supplementary material SM4) .

**Table 1:** Description of the sample (*n* = 2,087)

Category	*n*	*Mean (SD)*	*n (%) missing data*
Female	1274 (61.1%)	–	1 (< 1%)
Age	–	40.4 (13.5)	0
Education level, years	–	14.4 (2.7)	0
MADRS	–	4.2 (3.2)	141 (6.8%)
YMRS	–	2.2 (3.6)	140 (6.7%)
Type I BD	1088 (52.1%)	–	0
Type II BD	802 (38.4%)	–	0
Unspecified BD	197 (9.4%)	–	0
Current hypomanic episode	19 (< 1%)		92 (4.4%)
End of the last characterized mood episode > 3 months before	1725 (83.7%)	–	25 (1.2%)
Age at the first mood episode	–	24.4 (9.8)	175 (8.4%)
Duration of the bipolar disorder, years	–	9.0 (10.1)	164 (7.9%)
Number of depressive episodes	–	5.4 (5.9)	380 (18.2%)
Number of manic episodes	–	1.3 (2.1)	266 (12.7%)
Number of hypomanic episodes	–	3.4 (5.6)	593 (28.4%)
Patients with a history of psychosis	757 (42.2%)	–	294 (14.1%)
Number of medications	–	1.9 (1.3)	372 (17.8%)
Number of patients using antidepressants	487 (28.4%)	–	372 (17.8%)
…anticonvulsants	712 (41.5%)	–	372 (17.8%)
…lithium	504 (29.4%)	–	372 (17.8%)
…antipsychotics	627 (36.6%)	–	372 (17.8%)
…benzodiazepines	327 (19.1%)	–	372 (17.8%)
…anticholinergic agents	20 (1.2%)	–	372 (17.8%)
Anticholinergic burden, /24 h	–	0.66 (0.68)	372 (17.8%)
Chlorpromazine equivalents, mg/24 h	–	93.3 (206.2)	513 (24.6%)
Patients using the WAIS-IV version	1413 (67.7%)	–	0
Attention	–	−0.63 (1.37)	675 (32.3%)
Executive function	–	−0.24 (1.09)	49 (2.3%)
Working memory	–	−0.13 (0.94)	36 (1.7%)

**Table 2:** Association between manic symptom severity and attention performance in linear regression models estimated on complete cases

Main predictor		Raw estimates (n = 1,328)				Adjusted estimates (n = 1,130)			
		*Standardized β* (95% CI)	*p-value*	corr *p-value*^a^	*R*^*2*^	*Standardized β* (95% CI)	*p-value*	corr *p-value*^a^	*R*^*2*^
*Without a quadratic term*									
YMRS		−0.007 (−0.028, 0.015)	0.542	0.862	2.8.10^−4^	−0.007 (−0.030, 0.017)	0.584	0.877	0.07
Education level						**0.066 (0.038, 0.094)**	** < 0.001**	** < 0.001**	
BD subtype	Type I (ref)								
	Type II					0.159 (−0.017, 0.335)	0.066	0.246	
	Unspecified					0.094 (−0.202, 0.390)	0.526	0.858	
Medication use	Benzodiazepines					−0.026 (−0.226, 0.174)	0.799	0.968	
	Antidepressants					0.120 (−0.058, 0.298)	0.177	0.469	
	Lithium					**−0.403 (−0.581, −0.225)**	** < 0.001**	** < 0.001**	
	Other mood-stabilizers					0.017 (−0.145, 0.179)	0.838	0.967	
	Antipsychotics					**−0.246 (−0.412, −0.08)**	**0.003**	**0.019**	
Substance abuse						−0.130 (−0.456, 0.196)	0.426	0.804	
*With a quadratic term*									
YMRS		−0.005 (−0.057, 0.047)	0.851	0.985	3.9.10^−4^	0.002 (−0.031, 0.035)	0.911	0.967	0.07
YMRS^2^		−1.10^−4^ (−0.004, 0.004)	0.944	0.967		−0.044 (−0.149, 0.062)	0.417	0.799	
Education level						**0.067 (0.039, 0.095)**	** < 0.001**	** < 0.001**	
BD subtype	Type I (ref)								
	Type II					0.160 (−0.014, 0.334)	0.068	0.248	
	Unspecified					0.091 (−0.205, 0.387)	0.540	0.862	
Medication use	Benzodiazepines					−0.017 (−0.219, 0.185)	0.868	0.968	
	Antidepressants					0.111 (−0.069, 0.291)	0.217	0.538	
	Lithium					**−0.406 (−0.586, −0.226)**	** < 0.001**	** < 0.001**	
	Other mood-stabilizers					0.017 (−0.147, 0.181)	0.833	0.968	
	Antipsychotics					**−0.250 (−0.418, −0.082)**	**0.002**	**0.018**	
Substance abuse						−0.143 (−0.471, 0.185)	0.382	0.774	

^a^using false-discovery rate by Benjamini-Hochberg

Statistically significant associations are shown in bold

**Table 3:** Association between manic symptom severity and working memory in linear regression models (*n* = 2,087)

Main predictor		Raw estimates				Adjusted estimates			
		*Standardized β* (95% CI)	*p-value*	corr* p-value*^a^	*R*^*2*^	*Standardized β* (95% CI)	*p-value*	corr* p-value*^a^	*R*^*2*^
*Without a quadratic term*									
YMRS		−0.002 (−0.014, 0.01)	0.713	0.968	0.6.10^−4^	−0.004 (−0.017, 0.009)	0.505	0.842	0.09
Education level						**0.089 (0.073, 0.105)**	** < 0.001**	** < 0.001**	
BD subtype	Type I (ref)								
	Type II					0.069 (−0.031, 0.169)	0.166	0.456	
	Unspecified					0.208 (0.042, 0.374)	0.012	0.062	
Medication use	Benzodiazepines					−0.091 (−0.207, 0.025)	0.117	0.364	
	Antidepressants					0.026 (−0.076, 0.128)	0.609	0.894	
	Lithium					0.003 (−0.099, 0.105)	0.957	0.985	
	Other mood-stabilizers					−0.071 (−0.164, 0.022)	0.130	0.379	
	Antipsychotics					**−0.190 (−0.286, −0.094)**	** < 0.001**	** < 0.001**	
Substance abuse						0.137 (−0.048, 0.322)	0.139	0.397	
*With a quadratic term*									
YMRS		0.002 (−0.026, 0.029)	0.908	0.967	2.5.10^−4^	−0.003 (−0.021, 0.015)	0.724	0.967	0.09
YMRS^2^		−3.10^−4^ (−0.002, 0.002)	0.766	0.967		−0.006 (−0.060, 0.049)	0.837	0.967	
Education level						**0.085 (0.069, 0.101)**	** < 0.001**	** < 0.001**	
BD subtype	Type I (ref)								
	Type II					0.077 (−0.023, 0.177)	0.124	0.366	
	Unspecified					0.209 (0.043, 0.375)	0.012	0.067	
Medication use	Benzodiazepines					−0.082 (−0.198, 0.034)	0.158	0.448	
	Antidepressants					0.024 (−0.079, 0.127)	0.640	0.929	
	Lithium					−0.006 (−0.108, 0.096)	0.899	0.967	
	Other mood-stabilizers					−0.083 (−0.177, 0.011)	0.078	0.271	
	Antipsychotics					**−0.194 (−0.29, −0.098)**	** < 0.001**	** < 0.001**	
Substance abuse						0.120 (−0.066, 0.306)	0.199	0.507	

^a^using false-discovery rate by Benjamini-Hochberg

Statistically significant associations are shown in bold

**Table 4:** Association between manic symptom severity and executive function in linear regression models (*n* = 2,087)

Main predictor		Raw estimates				Adjusted estimates			
		*Standardized β* (95% CI)	*p-value*	corr* p-value*^a^	*R*^*2*^	*Standardized β* (95% CI)	*p-value*	corr* p-value*^a^	*R*^*2*^
*Without a quadratic term*									
YMRS		−1.10^−4^ (−0.014, 0.014)	0.987	0.993	0.5.10^−4^	−0.007 (−0.022, 0.009)	0.390	0.779	0.05
Education level						**0.060 (0.04, 0.08)**	** < 0.001**	** < 0.001**	
BD subtype	Type I (ref)								
	Type II					**0.254 (0.138, 0.37)**	** < 0.001**	** < 0.001**	
	Unspecified					0.227 (0.033, 0.421)	0.019	0.098	
Medication use	Benzodiazepines					−0.143 (−0.279, −0.007)	0.037	0.152	
	Antidepressants					−0.047 (−0.167, 0.073)	0.435	0.816	
	Lithium					**−0.272 (−0.39, −0.154)**	** < 0.001**	** < 0.001**	
	Other mood-stabilizers					−0.022 (−0.132, 0.088)	0.688	0.967	
	Antipsychotics					−0.076 (−0.188, 0.036)	0.176	0.468	
Substance abuse						−0.091 (−0.309, 0.127)	0.405	0.796	
*With a quadratic term*									
YMRS		0.003 (−0.029, 0.034)	0.878	0.967	2.3.10^−4^	−0.019 (−0.041, 0.003)	0.082	0.276	0.06
YMRS^2^		−2.10^−4^ (−0.003, 0.002)	0.862	0.967		0.057 (−0.008, 0.121)	0.084	0.276	
Education level						**0.061 (0.041, 0.081)**	** < 0.001**	** < 0.001**	
BD subtype	Type I (ref)								
	Type II					**0.242 (0.127, 0.359)**	** < 0.001**	** < 0.001**	
	Unspecified					0.236 (0.041, 0.431)	0.015	0.081	
Medication use	Benzodiazepines					−0.144 (−0.28, −0.008)	0.036	0.153	
	Antidepressants					−0.046 (−0.168, 0.076)	0.453	0.823	
	Lithium					**−0.286 (−0.407, −0.165)**	** < 0.001**	** < 0.001**	
	Other mood-stabilizers					−0.028 (−0.138, 0.082)	0.608	0.894	
	Antipsychotics					−0.078 (−0.191, 0.035)	0.169	0.460	
Substance abuse						−0.114 (−0.332, 0.104)	0.301	0.646	

^a^using false-discovery rate by Benjamini-Hochberg

Statistically significant associations are shown in bold

Among covariates, higher education level was significantly associated with better cognitive performance even after adjusting *p*-values for multiple comparisons (Tables 2, 3, and 4). Additionally, attention performance was significantly lower in lithium or antipsychotic users. Working memory was significantly better when using the WAIS-IV version (supplementary material SM1), and worse in antipsychotic users. Executive function was significantly better in people with type II BD and worse in lithium users.

## Discussion

We found no significant quadratic associations between hypomanic symptom severity and attention, working memory, and executive function in a large sample of adults with BD. Consequently, we were unable to replicate the quadratic association reported by Koenders and collaborators between YMRS score and attention, despite the similarities between our studies’ design, exclusion criteria, and covariates.

Several methodological differences between the two studies may account for the discrepant findings. First, we did not directly measure divided attention, but alternative measures of sustained attention, executive function and working memory. The divided attention subtest of the TAP may be more sensitive to mania-induced attentional improvements but further evidence is needed. Second, Koenders and collaborators used a within-patient design, whereas the present study is cross-sectional with one measure per subject. The precision of measurement may thus be lower in our study. Yet, our larger sample size likely compensated for this. Unlike Koenders and collaborators, we were unable to adjust for actual substance use, such as tobacco or alcohol consumption, factors known to affect manic symptoms and cognition (Strakowski et al. 2005). However, we adjusted for the occurrence of symptoms of substance use disorders in the month preceding the visit, which likely accounted for the majority of patients with recent intake. Moreover, Koenders and collaborators did not exclude individuals experiencing a current depressive episode during cognitive testing. This inclusion may explain why patients with the lowest manic symptom severity, who were actually depressed, exhibited poorer divided attention than those with mild manic symptoms. Consequently, this may have led to a spurious quadratic association between manic symptom severity and cognitive performance. These methodological differences may explain our inability to replicate the original study’s main effect.

However, similarly to Koenders and collaborators, the linear association between the total YMRS score and attention was non-significant. These results differ from those reported by Zhang and collaborators, who found a linear association between residual manic symptoms and worse memory and attention. Individuals in our study had low manic symptom severity like in Zhang and collaborators’ study. The discrepancy might be explained by the difference in the scales as those authors used the BRMS, which, unlike YMRS, does not evaluate insight. Besides, they only adjusted for education level in their regression models, which may have led to an overestimation of the effect. When investigating the impact of hypomanic symptoms on cognitive performance, it is crucial to account for established risk factors for cognitive impairment, such as age, illness duration, medication, and comorbidities (Gogia et al. 2022; Roux et al. 2019; Vidal et al. 2023). These variables are often associated with both the severity of manic symptoms and an individual’s overall cognitive health. Failing to control for them may confound the observed association.

A major strength of our study is the large sample of patients with a DSM-verified diagnosis of BD, supported by a collection of valid and reliable measurements of cognitive abilities. Another important strength of the study is the exclusion of individuals with residual depressive symptoms, which may have obscured the association between hypomania and cognition. Additionally, our comprehensive database enabled us to investigate the influence of various clinical variables, such as a history of psychosis, the number of manic episodes, and medication use, on the associations between YMRS scores and cognitive performance. Limitations of our study include the low variance and mean of manic symptom severity, which may explain the absence of significant associations. Because our participants exhibited very few hypomanic symptoms, it may have been difficult to capture an effect, particularly a quadratic (inverted U-shaped) association, which requires data from patients with high symptom severity to complete the "bell-shaped" curve. Besides, we did not assess social cognitive performance, which is often considerably impaired in BD. The effects of mild hypomanic symptoms on social cognition require further investigation (Samamé et al. 2012).

Overall, our results support that mild manic symptoms have no or negligible effects on divided attention in BD, calling into question the functional benefit of residual manic symptoms. These findings suggest that euthymic patients with residual mild hypomanic symptoms should not be excluded from pro-cognitive clinical trials. In clinical settings, treating the residual mild hypomanic symptoms of euthymic patients is unlikely to improve their cognitive performance. Instead, we recommend prioritizing other strategies, such as searching for a modifiable primary cause of cognitive deficit (comorbid ADHD, mood stabilizer overdosage, dysthyroidism, benzodiazepine usage, etc.), and if such a cause is lacking, prioritize an intervention like cognitive remediation.

## Supplementary Information

Additional file 1.

## Data Availability

Due to ethical and legal restrictions, data involving clinical participants cannot be made publicly available. All relevant data are available upon request to the Fondation FondaMental for researchers who meet the criteria for access to confidential data.

## Abbreviation

BD: Bipolar disorders
