# Supplementary material for: Examining the associations between manic symptoms and cognitive performance in bipolar disorders: evidence from a cross-sectional replication study in the FACE-BD cohort
Source: Int J Bipolar Disord. 2026 Apr 15;14:14. doi: 10.1186/s40345-026-00420-2 (PMC13083468; doi:10.1186/s40345-026-00420-2)
Supplement: Supplementary file 1 — Additional file 1. [file 40345_2026_420_MOESM1_ESM.docx]

# Supplementary material

Examining the Associations between Manic Symptoms and Cognitive Performance in Bipolar Disorders: Evidence from a Cross-sectional Replication Study in the FACE-BD cohort

[**Supplementary material 1**](#_d53q6p7p3hfa)

[SM1: Association between manic symptom severity and cognition performance in multiple models adjusting for additional covariates. 2](#_t41gz7t8ymgs)

[SM2: Association between manic symptom severity and cognitive performance after excluding individuals aged >65 years. 4](#_23v45yvuk4xi)

[SM3: Association between manic symptom severity and cognitive performance after excluding individuals with a global cognitive performance score within two standard deviations of the mean, after a within-sample standardisation. 5](#_5zk56isuh6yh)

[SM4: Association between manic symptom severity and attention performance across subdomains of the YMRS scale in multiple linear regression models. 6](#_otfdzvf6om3n)

### SM1: Association between manic symptom severity and cognition performance in multiple models adjusting for additional covariates.

| **Predictor** | | **Adjusted estimates^a^** | | | |
| --- | --- | --- | --- | --- | --- |
|  |  |  |  |  |  |
|  |  | ***Standardized β***  **(95% CI)** | ***p-value*** | **corr *p-value***^b^ | ***R^2^*** |
|  |  |  |  |  |  |
| **Attention (*n* = 796)** | | | | | |
| **Without a quadratic term** | | | | | |
| YMRS | | 0.0023 (-0.0263, 0.0309) | .873 | .968 | 0.06 |
| Anticholinergic burden | | -0.076 (-0.246, 0.094) | .369 | .611 |  |
| History of psychosis | | 0.086 (-0.184, 0.356) | .524 | .857 |  |
| Duration of the bipolar disorder | | 0.008 (-0.004, 0.02) | .211 | .799 |  |
| WAIS | | -0.115 (-0.321, 0.091) | .267 | .611 |  |
| Numbers of previous manic episodes | | -0.011 (-0.03, 0.008) | .254 | .500 |  |
| Last manic episode occurred within 3 months | | -0.268 (-1.082, 0.546) | .511 | .662 |  |
| **With a quadratic term** | | | | | |
| YMRS | | -0.0012 (-0.0418, 0.0395) | .954 | .986 | 0.06 |
| YMRS^2^ | | 0.0142 (-0.1088, 0.1372) | .820 | .968 |  |
| Anticholinergic burden | | -0.078 (-0.248, 0.092) | .358 | .600 |  |
| History of psychosis | | 0.079 (-0.193, 0.351) | .559 | .858 |  |
| Duration of the bipolar disorder | | 0.008 (-0.004, 0.020) | .219 | .805 |  |
| WAIS | | -0.104 (-0.312, 0.104) | .319 | .612 |  |
| Numbers of previous manic episodes | | -0.012 (-0.031, 0.007) | .227 | .456 |  |
| Last manic episode occurred within 3 months | | -0.276 (-1.092, 0.54) | .499 | .646 |  |
| **Executive function (*n* = 2,087)** | | | | | |
| **Without a quadratic term** | | | | | |
| YMRS | | -0.003 (-0.022, 0.016) | .746 | .967 | 0.08 |
| Anticholinergic burden | | -0.103 (-0.215, 0.009) | .066 | .121 |  |
| History of psychosis | | 0.121 (-0.053, 0.295) | .168 | .839 |  |
| Duration of the bipolar disorder | | 0.001 (-0.007, 0.009) | .734 | .967 |  |
| WAIS | | 0.111 (-0.027, 0.249) | .110 | .153 |  |
| Numbers of previous manic episodes | | -0.015 (-0.027, -0.003) | .010 | .081 |  |
| Last manic episode occurred within 3 months | | 0.083 (-0.493, 0.659) | .772 | .967 |  |
| **With a quadratic term** | | | | | |
| YMRS | | -0.0100 (-0.0372, 0.0172) | 0.469 | .828 | 0.085 |
| YMRS^2^ | | 0.0314 (-0.0509, 0.1137) | 0.453 | .823 |  |
| Anticholinergic burden | | -0.105 (-0.217, 0.007) | .062 | .106 |  |
| History of psychosis | | -0.116 (-0.293, 0.061) | .192 | .864 |  |
| Duration of the bipolar disorder | | 0.001 (-0.007, 0.009) | .766 | .968 |  |
| WAIS | | 0.124 (-0.016, 0.264) | .777 | .147 |  |
| Numbers of previous manic episodes | | -0.015 (-0.027, -0.003) | .015 | .101 |  |
| Last manic episode occurred within 3 months | | 0.079 (-0.500, 0.657) | .784 | .968 |  |
| **Working memory (*n* = 2,087)** | | | | | |
| **Without a quadratic term** | | | | | |
| YMRS | | -0.0025 (-0.0187, 0.0136) | .756 | .967 | 0.11 |
| Anticholinergic burden | | -0.023 (-0.124, 0.078) | .646 | .857 |  |
| History of psychosis | | -0.030 (-0.191, 0.131) | .705 | .968 |  |
| Duration of the bipolar disorder | | 0.005 (-0.002, 0.012) | .200 | .602 |  |
| WAIS | | **0.374 (0.25, 0.498)** | **<.001** | **<.001** |  |
| Numbers of previous manic episodes | | -0.004 (-0.015, 0.007) | .413 | .588 |  |
| Last manic episode occurred within 3 months | | 0.102 (-0.392, 0.596) | .680 | .968 |  |
| **With a quadratic term** | | | | | |
| YMRS | | -0.0035 (-0.0268, 0.0197) | .764 | .967 | 0.11 |
| YMRS^2^ | | 0.0038 (-0.0674, 0.0750) | .916 | .967 |  |
| Anticholinergic burden | | -0.028 (-0.129, 0.073) | .584 | .828 |  |
| History of psychosis | | -0.013 (-0.175, 0.149) | .875 | .987 |  |
| Duration of the bipolar disorder | | 0.004 (-0.004, 0.012) | .239 | .646 |  |
| WAIS | | **0.370 (0.244, 0.495)** | **<.001** | **<.001** |  |
| Numbers of previous manic episodes | | -0.002 (-0.013, 0.009) | .767 | .836 |  |
| Last manic episode occurred within 3 months | | 0.106 (-0.389, 0.602) | .668 | .968 |  |

###

###

### SM2: Association between manic symptom severity and cognitive performance after excluding individuals aged >65 years.

| **Attention** | | | | | | | | | |
| --- | --- | --- | --- | --- | --- | --- | --- | --- | --- |
| **Main predictor** | | **Raw estimates (n = 1,328)** | | | | **Adjusted estimates^a^ (n = 1,130)** | | | |
|  |  |  |  |  |  |  |  |  |  |
|  |  | ***Standardized β***  **(95% CI)** | ***p-value*** | **corr *p-value***^b^ | ***R^2^*** | ***Standardized β***  **(95% CI)** | ***p-value*** | **corr *p-value***^b^ | ***R^2^*** |
|  |  |  |  |  |  |  |  |  |  |
| **Without a quadratic term** | | | | | | | | | |
| YMRS | | -0.004 (-0.026, 0.017) | 0.693 | .967 | 1.10^-4^ | -0.002 (-0.032, 0.015) | 0.495 | .839 | 0.07 |
| **With a quadratic term** | | | | | | | | | |
| YMRS | | -0.001 (-0.052, 0.05) | 0.963 | .986 | 1.2.10^-4^ | 0.004 (-0.030, 0.037) | 0.838 | .967 | 0.07 |
| YMRS^2^ | | -3.10^-4^ (-0.004, 0.004) | 0.895 | .967 |  | -0.056 (-0.165, 0.053) | 0.315 | .668 |  |
| **Executive function (*n* = 1,995)** | | | | | | | | | |
| **Main predictor** | | **Raw estimates** | | | | **Adjusted estimates^a^** | | | |
|  |  |  |  |  |  |  |  |  |  |
|  |  | ***Standardized β***  **(95% CI)** | ***p-value*** | **corr *p-value***^b^ | ***R^2^*** | ***Standardized β***  **(95% CI)** | ***p-value*** | **corr *p-value***^b^ | ***R^2^*** |
|  |  |  |  |  |  |  |  |  |  |
| **Without a quadratic term** | | | | | | | | | |
| YMRS | | 0.001 (-0.013, 0.015) | 0.888 | .967 | 0.7.10^-4^ | -0.007 (-0.023, 0.008) | 0.345 | .714 | 0.06 |
| **With a quadratic term** | | | | | | | | | |
| YMRS | | 0.003 (-0.029, 0.035) | 0.872 | .967 | 3.1.10^-4^ | -0.017 (-0.039, 0.005) | 0.121 | .366 | 0.06 |
| YMRS^2^ | | -1.10^-4^ (-0.002, 0.002) | 0.910 | .967 |  | 0.042 (-0.024, 0.109) | 0.213 | .533 |  |
| **Working memory (*n* = 1,995)** | | | | | | | | | |
| **Main predictor** | | **Raw estimates** | | | | **Adjusted estimates^a^** | | | |
|  |  |  |  |  |  |  |  |  |  |
|  |  | ***Standardized β***  **(95% CI)** | ***p-value*** | **corr *p-value***^b^ | ***R^2^*** | ***Standardized β***  **(95% CI)** | ***p-value*** | **corr *p-value***^b^ | ***R^2^*** |
|  |  |  |  |  |  |  |  |  |  |
| **Without a quadratic term** | | | | | | | | | |
| YMRS | | -0.002 (-0.014, 0.01) | 0.716 | .967 | 0.2.10^-4^ | -0.005 (-0.018, 0.008) | 0.470 | .827 | 0.08 |
| **With a quadratic term** | | | | | | | | | |
| YMRS | | 0.001 (-0.027, 0.029) | 0.942 | .985 | 3.6.10^-4^ | -0.003 (-0.021, 0.016) | 0.790 | .967 | 0.08 |
| YMRS^2^ | | -3.10^-4^ (-0.002, 0.002) | 0.799 | .967 |  | -0.011 (-0.067, 0.046) | 0.712 | .967 |  |

###

### SM3: Association between manic symptom severity and cognitive performance after excluding individuals with a global cognitive performance score within two standard deviations of the mean, after a within-sample standardisation.

| **Attention** | | | | | | | | | |
| --- | --- | --- | --- | --- | --- | --- | --- | --- | --- |
| **Main predictor** | | **Raw estimates (n = 1,265)** | | | | **Adjusted estimates^a^ (n = 1,073)** | | | |
|  |  |  |  |  |  |  |  |  |  |
|  |  | ***Standardized β***  **(95% CI)** | ***p-value*** | **corr *p-value***^b^ | ***R^2^*** | ***Standardized β***  **(95% CI)** | ***p-value*** | **corr *p-value***^b^ | ***R^2^*** |
|  |  |  |  |  |  |  |  |  |  |
| **Without a quadratic term** | | | | | | | | | |
| YMRS | | -0.002 (-0.024, 0.019) | 0.8479 | .967 | 1.1.10^-4^ | -0.001 (-0.025, 0.023) | 0.926 | .975 | 0.07 |
| **With a quadratic term** | | | | | | | | | |
| YMRS | | -7.10^-4^ (-0.053, 0.052) | 0.978 | .992 | 3.6.10^-4^ | 0.004 (-0.029, 0.036) | 0.833 | .967 | 0.07 |
| YMRS^2^ | | -1.10^-4^ (-0.004, 0.004) | 0.957 | .985 |  | -0.026 (-0.134, 0.083) | 0.643 | .929 |  |
| **Executive functions (*n* = 1,966)** | | | | | | | | | |
| **Main predictor** | | **Raw estimates** | | | | **Adjusted estimates^a^** | | | |
|  |  |  |  |  |  |  |  |  |  |
|  |  | ***Standardized β***  **(95% CI)** | ***p-value*** | **corr *p-value***^b^ | ***R^2^*** | ***Standardized β***  **(95% CI)** | ***p-value*** | **corr *p-value***^b^ | ***R^2^*** |
|  |  |  |  |  |  |  |  |  |  |
| **Without a quadratic term** | | | | | | | | | |
| YMRS | | 0.002 (-0.011, 0.015) | 0.748 | .967 | 7.4.10^-5^ | -0.003 (-0.018, 0.011) | 0.640 | .929 | 0.06 |
| **With a quadratic term** | | | | | | | | | |
| YMRS | | 0.015 (-0.014, 0.044) | 0.320 | .670 | 3.6.10^-4^ | -0.011 (-0.031, 0.009) | 0.2993 | .646 | 0.05 |
| YMRS^2^ | | -0.001 (-0.003, 0.001) | 0.346 | .714 |  | 0.033 (-0.029, 0.095) | 0.296 | .646 |  |
| **Working memory (*n* = 1,966)** | | | | | | | | | |
| **Main predictor** | | **Raw estimates** | | | | **Adjusted estimates^a^** | | | |
|  |  |  |  |  |  |  |  |  |  |
|  |  | ***Standardized β***  **(95% CI)** | ***p-value*** | **corr *p-value***^b^ | ***R^2^*** | ***Standardized β***  **(95% CI)** | ***p-value*** | **corr *p-value***^b^ | ***R^2^*** |
|  |  |  |  |  |  |  |  |  |  |
| **Without a quadratic term** | | | | | | | | | |
| YMRS | | -0.003 (-0.015, 0.008) | 0.566 | .877 | 2.0.10^-4^ | -0.005 (-0.017, 0.008) | 0.484 | .836 | 0.08 |
| **With a quadratic term** | | | | | | | | | |
| YMRS | | 0.007 (-0.02, 0.034) | 0.597 | .889 | 2.1.10^-4^ | 0.001 (-0.017, 0.019) | 0.912 | .967 | 0.08 |
| YMRS^2^ | | -9.10^-4^ (-0.003, 0.001) | 0.394 | .780 |  | -0.026 (-0.081, 0.029) | 0.348 | .714 |  |

###

### SM4: Association between manic symptom severity and attention performance across subdomains of the YMRS scale in multiple linear regression models.

| **Main predictor** | ***Adjusted β* (95% CI)** | ***p-value*** | **corrected *p-value*^a^** | ***R^2^*** |
| --- | --- | --- | --- | --- |
|  |  |  |  |  |
| YMRS01: elevated mood | -0.0454 (-0.4446, 0.3539) | 0.823 | .967 | 0.07 |
| YMRS01^2^ | -0.0163 (-0.2334, 0.2007) | 0.882 | .967 |  |
| YMRS02: increased motor activity | 0.1461 (-0.2237, 0.5159) | 0.438 | .816 | 0.07 |
| YMRS02^2^ | -0.0837 (-0.2642, 0.0968) | 0.363 | .739 |  |
| YMRS03: sexual interest | -0.7973 (-1.4387, -0.1559) | **0.014** | .079 | 0.08 |
| YMRS03^2^ | 0.3692 (0.0065, 0.7318) | **0.046** | .188 |  |
| YMRS04: sleep | 0.2084 (-0.1771, 0.5940) | 0.289 | .646 | 0.07 |
| YMRS04^2^ | -0.0578 (-0.2165, 0.1009) | 0.475 | .830 |  |
| YMRS05: irritability | -0.1171 (-0.3180, 0.0838) | 0.253 | .600 | 0.07 |
| YMRS05^2^ | 0.0406 (-0.0205, 0.1018) | 0.192 | .500 |  |
| YMRS06: speech | 0.0160 (-0.1910, 0.2229) | 0.879 | .967 | 0.07 |
| YMRS06^2^ | 0.0003 (-0.0515, 0.0522) | 0.989 | .993 |  |
| YMRS07: language-thought disorder | -0.3556 (-0.9142, 0.2030) | 0.2119 | .533 | 0.07 |
| YMRS07^2^ | 0.2148 (-0.1551, 0.5847) | 0.254 | .600 |  |
| YMRS08: content | -0.0013 (-0.5522, 0.5497) | 0.996 | .992 | 0.07 |
| YMRS08^2^ | 0.0029 (-0.2110, 0.2169) | 0.978 | .996 |  |
| YMRS09: disruptive-agressive behavior | 0.5325 (-0.3560, 1.4211) | 0.239 | .366 | 0.08 |
| YMRS09^2^ | -0.2839 (-0.6436, 0.0758) | 0.121 | .585 |  |
| YMRS10: appearance | -0.3129 (-1.7387, 1.1129) | 0.666 | .957 | 0.07 |
| YMRS10^2^ | 0.1901 (-0.8813, 1.2614) | 0.727 | .967 |  |
| YMRS11: insight | -0.1334 (-0.5949, 0.3280) | 0.570 | .877 | 0.07 |
| YMRS11^2^ | 0.0221 (-0.1624, 0.2066) | 0.814 | .967 |  |

^a^false-discovery rate by Benjamini-Hochberg
